# Editorial: Empowering patients and supporting patient-centered care: a spotlight on health behavior change

**DOI:** 10.3389/fmed.2024.1474005

**Published:** 2024-08-30

**Authors:** Christos Lionis, Sophia Papadakis, Marilena Anastasaki, Ana M. Carriazo

**Affiliations:** ^1^Laboratory of Health and Society, School of Medicine, University of Crete, Crete, Greece; ^2^Junta de Andalucia, Sevilla, Spain

**Keywords:** empower, support, patient-centered care, health behavior (MeSH), change

The COVID-19 pandemic has had a major impact on both mental and physical health, with documented and ongoing effects on the health status of populations globally (1–6). Some of the drivers of these trends include the rise in risk factors and unhealthy behaviors in the population (e.g., obesity, smoking, and alcohol consumption); poor adherence to health screening, vaccination, or other preventative interventions; and increased rates of mental illness (4–13). Importantly, the greatest effects have been documented in the most vulnerable populations (3, 6, 14).

The pandemic's effects highlight the need for actions targeted toward disease prevention, with a focus on health behavior change. It is recognized that such interventions should be grounded in the principles of person-centered approaches, including patient empowerment and compassionated care provision, in order to address the complex interactions between mental and physical health and promote effective communication between healthcare professionals and patients (15–17). Sharing international learning and best practice for promoting health behavior change is key to supporting the rapid scale-up of effective intervention strategies.

This Frontiers in Medicine Research Topic “*Empowering Patients and Supporting Patient-Centered Care: A Spotlight on Health Behavior Change*” sought to collect the best and most promising empowerment-oriented strategies for supporting health behavior change. One of our key ambitions for this Research Topic was the examination of methods that address individuals, populations, and healthcare professionals and aim to reduce the risk of disease, promote healthy behaviors, or enhance adherence to healthcare interventions. The four themes that this topic aimed to include were

1. interventions and initiatives to address chronic disease risk factors including smoking cessation, alcohol use reduction, and physical exercise improvement,
2. interventions and initiatives to improve self-management and care of chronic diseases, including adherence to medicine and other therapies,
3. interventions and initiatives to prevent communicable/infectious diseases and associated behaviors, such as vaccination and other preventative interventions and policies,
4. experience and interventions focused on transferring effective interventions into complex settings and contexts focusing on multimorbidity and frailty.

We had the pleasure of communicating with many research teams and, ultimately, a collection of nine manuscripts has been published as part of this Research Topic.

Improving patient-centered mental health promotion in primary health care (PHC) to support vulnerable communities through mindfulness training was the focus of a Brazilian intervention by Teixeira et al.. Their intervention was based on the Mindfulness-Based Health Promotion Model, which promotes self-care and psychological support in PHC. PHC and self-care were also the focus of a systematic review by Rakers et al., which demonstrated that population health management (PHM)-related interventions can reach many participants and are effective in reducing cardiometabolic risk factors. Self-management and self-care are among the most challenging issues that PHC and public health practitioners have to address to achieve the United Nations Sustainable Development Goal #3 “Ensuring a healthy life and promoting wellbeing for all ages” (18). The role of caregivers in achieving this target is also vital and the WHO Astana Declaration (2018) emphasizes the need to turn our attention to this group of care providers (19). Mas-Casadesus et al., in their original article, underline the need for policymakers to introduce community-based and planned interventions aimed at caregivers to improve the management of vulnerable people during periods of isolation. The findings are particularly relevant given the isolation that was experienced by a large number of people and caregivers during and after the pandemic. A perspective article by Cipta et al. reported on the impact of integrating culture-specific patient empowerment practices into healthcare settings in Indonesia. This article underscores the potential for improved health outcomes, heightened patient engagement, and the delivery of cultural services within low and middle-income countries.

This Research Topic includes two articles that address COVID-19 vaccine hesitancy, a subject that is particularly relevant given the challenges reported worldwide in achieving national vaccination program targets (3, 20, 21). Papadakis et al., reported on the development and pilot testing of an eLearning intervention for PHC practitioners and social care providers to reduce vaccination hesitancy among patients in Greece. The intervention sought to develop training on how Very Brief Advice (VBA) and motivational interviewing (MI) can be adapted to promote COVID-19 vaccine uptake and address ambivalence and resistance among patients. In a similar direction, Lorenzo et al., in their policy report, stressed the need for effective communication strategies to tackle vaccination hesitancy. They clearly underlined that trained professionals should curate communication with the public.

Saeed et al., in their original research, reported the level of satisfaction among COVID-19 survivors and discussed the challenges of healthcare affordability and the role of healthcare practitioners in Northeast India. They consider the challenges in healthcare affordability and timeliness as important. The challenge of value-based primary care, which measures improvement in patient health outcomes relative to the cost of achieving that improvement, was the focus of a article by Rangachari. This article examines how healthcare consumerism can act as a barrier or facilitate the implementation of value-based primary care.

Fernandes et al., in their study protocol, presented the key elements of a feasibility study for Parkinson's disease. Apart from its focus on Parkinson's as a growing health concern, this small study provided insights into the design of a community-based intervention that encompasses elements of group cognitive behavioral therapy in addition to disease management and training techniques. Such interventions in the community addressing chronic illness and disability with a focus on behavior and mental health may offer important lessons for enabling and empowering patients through health behavior support.

We hope the collection of articles featured in this Research Topic will give prominence to the importance of patient-centered approaches in improving self-care and facilitating behavior change. We are pleased to be able to share this collection of articles with the field and hope it serves to inform and inspire practitioners, policymakers, and researchers on the importance of patient-centered models to health behavior change as we continue to address the health of populations with a new perspective and insight in the post-pandemic period, with an eye to future potential health crises.
